# Virus Log Reduction Values and Dominant Mechanisms in Full‐Scale Secondary Biological Wastewater Treatment Systems

**DOI:** 10.1002/wer.70180

**Published:** 2025-09-18

**Authors:** Phillip Wang, Tyler Hill, Christina Morrison, Katherine Crank, Jacimaria Batista, Daniel Gerrity

**Affiliations:** ^1^ Southern Nevada Water Authority Las Vegas Nevada USA; ^2^ Department of Civil and Environmental Engineering and Construction University of Nevada Las Vegas Las Vegas Nevada USA; ^3^ Black & Veatch Las Vegas Nevada USA

**Keywords:** activated sludge, direct potable reuse (DPR), lagoon, pathogen, potable reuse, qPCR

## Abstract

Secondary biological wastewater treatment is a core component of potable reuse treatment trains, but it is often uncredited for virus attenuation. This study evaluated virus log reduction values (LRVs) at two full‐scale water resource recovery facilities in Southern Nevada: one with conventional activated sludge and another with lagoon treatment. Four human enteric viruses and four fecal indicator viruses were quantified using molecular assays; culture assays quantified F‐specific and somatic coliphages. Median LRVs were low (< 1.0) for plant viruses and generally higher (> 1.0) for adenovirus and crAssphage, consistent with predictions from a solids partitioning model. The fifth percentile LRVs that often drive regulatory determinations were < 0.5 for norovirus GI/GII. For conventional activated sludge, nucleic acid decay was a significant contributor to the molecular LRVs, whereas culturable coliphage data (median LRV = 2.5) highlighted solids attachment and subsequent physical removal as a dominant mechanism. In contrast, lagoon treatment sometimes achieved LRVs > 4.0 for culturable coliphages, primarily due to temperature‐dependent inactivation. Based on the collective insight from recent studies, adoption of a broad virus LRV crediting framework for conventional activated sludge systems in potable reuse applications will require better alignment between molecular and culture methods and a deeper understanding of virus attenuation mechanisms.

## Summary

- The dominant LRV mechanism varied based on the method of virus quantification and the type of secondary treatment system.
- For conventional activated sludge, mean molecular LRVs ranged from 0.8 to 2.6, but fifth percentile LRVs for norovirus were < 0.5.
- Non‐specific culturing of coliphages resulted in mean LRVs ≥ 2.5 for the conventional activated sludge and lagoon systems.
- Use of molecular methods or culturable coliphages as surrogates for infectious enteric viruses may not adequately protect public health.
- Modified molecular methods and a deeper understanding of mechanisms are needed for the adoption of virus LRV crediting for secondary treatment.

## Graphical abstract

For secondary treatment, the dominant virus removal mechanism varies based on system type and method(s) used to quantify virus reduction. This study demonstrates that secondary treatment can achieve significant virus reduction, but regulatory crediting for direct potable reuse may still not be justified.

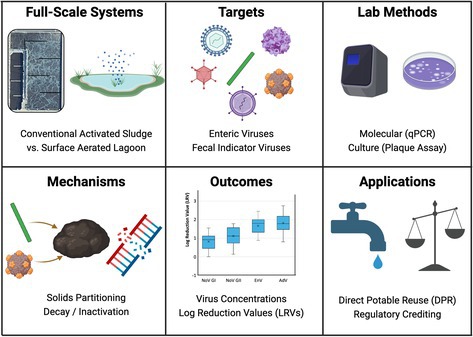

## 1 Introduction

Potable reuse seeks to address the growing challenge of water scarcity and the need for sustainable water supplies across the United States. Considering that the product water is intended for human consumption, potable reuse regulatory frameworks are often considerably more stringent than those for other recycled water applications (EPA 2012, 2017). Moreover, because direct potable reuse (DPR) systems lack the additional response retention time afforded by indirect potable reuse (IPR), DPR regulatory frameworks sometimes include additional safety factors and more extensive compliance criteria. In the United States, these potable reuse regulations are developed at the state level, with each state establishing its own pathogen log reduction value (LRV) targets as part of a multi‐barrier treatment approach.

Viruses present a challenge for potable reuse due to their prevalence in raw wastewater and their public health relevance at concentrations well below the detection limits of conventional analytical methods. With the goal of achieving an acceptable drinking water concentration on the order of 1 virus per 10 million liters, LRV targets for DPR range from 8 log_10_ in Texas (at a minimum) to 12–14 log_10_ in Colorado, Arizona, and Florida, and 20 log_10_ in California (Gerrity et al. 2023). This disparity across states stems from differences in the underlying assumptions used in their risk assessment frameworks. For example, the starting point for LRV crediting in Texas is treated wastewater effluent rather than raw wastewater and is based on site‐specific monitoring. On the other hand, California assumes worst‐case scenarios for raw wastewater pathogen concentrations and treatment failure, leading to a more conservative approach and higher LRV targets. Regardless of these underlying differences, all DPR frameworks demand extensive treatment trains to accumulate sufficient LRV credits for regulatory compliance.

Each unit process in a treatment train can potentially contribute to pathogen reduction, but there are certain treatment processes that may be uncredited or undercredited in potable reuse applications (Amoueyan et al. 2019; Polanco et al. 2023), either due to variability in performance (Hill et al. 2025) or inadequate surrogates (Ray et al. 2024). One such process is secondary biological treatment, which relies on an activated sludge system to remove biochemical oxygen demand (BOD) and nutrients. Secondary biological treatment also contributes to virus attenuation through mechanisms such as adsorption, predation, and natural inactivation (Kim et al. 1995; Wen et al. 2009; Worley‐Morse et al. 2019), but relationships between virus attenuation and operational or water quality conditions remain poorly understood. Thus, secondary biological treatment currently lacks a robust surrogate framework, which is a key component of any critical control point in a potable reuse system (Walker et al. 2020). This hinders regulatory crediting and highlights the need for further research to identify the dominant factors driving virus attenuation.

A recent review of conventional activated sludge systems highlighted the wide range of reported virus LRVs in the literature and noted that there was little overlap across studies, making it difficult to draw broad conclusions (Hill et al. 2025). However, Wang et al. (2025) successfully demonstrated the role of solids retention time (SRT) in virus removal, specifically via increases in mixed liquor suspended solids (MLSS) concentrations. However, that study's proposed model for predicting virus LRVs was developed at bench scale, thereby warranting further validation in large‐scale systems before it can be considered for any crediting framework. To address this need, the current study measured virus LRVs at two full‐scale water resource recovery facilities (WRRFs) in Southern Nevada, with one operating a conventional activated sludge system and the other operating a lagoon system. Culture targets included F‐specific and somatic coliphages, and molecular targets included four human enteric viruses (adenovirus [AdV], enterovirus [EnV], norovirus [NoV] GI, and NoV GII) and four fecal indicator viruses (pepper mild mottle virus [PMMoV], cucumber green mottle mosaic virus [CGMMV], MS2 bacteriophage, and crAssphage [*Carjivirus communis*]). The data were evaluated against the historical literature, including the aforementioned bench‐scale study and its MLSS‐based model for predicting virus LRVs. Through systematic adjustments to sample processing and analysis, this study also explored dominant virus reduction mechanisms in conventional activated sludge versus lagoon systems. Ultimately, this information may contribute to future decision‐making efforts for the permitting, design, and implementation of potable reuse, particularly DPR.

## 2 Methods

### 2.1 Study Sites

Samples were collected from two full‐scale WRRFs in Southern Nevada (Facility 1 and Facility 2). Facility 1 treats ~100 million gallons per day (mgd) with a conventional activated sludge system operated at an SRT of ~7 days. Following primary clarification supplemented with a ferric chloride dose of 6 mg/L, secondary biological treatment targets BOD removal, full nitrification, partial denitrification, and biological phosphorus removal using a modified Johannesburg process with a series of anoxic, anaerobic, and aerobic zones. Facility 2 treats ~1 mgd with two parallel trains, each consisting of a surface‐aerated complete mix lagoon (CML) with a hydraulic retention time (HRT) of ~2 days and a series of surface‐aerated partial mix lagoons (PMLs) with a combined HRT of ~5 days. Schematics of these facilities are provided in Figure S1.

At Facility 1, samples from the combined aeration basin influent (i.e., primary effluent [PE]) and combined secondary effluent (SE) were collected 3 days per week over 12 consecutive weeks from August to November 2022 (*N* = 36 paired composite samples). These samples were collected on Sunday, Monday, and Tuesday mornings as 24‐h composites using a refrigerated Hach AS950 autosampler (Loveland, CO). The average daily air temperature across the sampling period ranged from highs of 28°C–37°C in August to lows of 7°C–19°C in November. In addition, grab samples were collected from seven different zones within a single activated sludge basin on three separate occasions (*N* = 21 total grab samples). These grab samples were collected in November and December of 2022; over this time period, the average daily air temperature ranged from 4°C to 14°C. The seven zones included (in order) the anoxic return activated sludge (RAS) inlet, the anaerobic PE inlet, another anaerobic zone, three sequential aerobic zones, and the aerobic basin outfall (Figure S1). The intent of these grab samples was to assess virus attenuation mechanisms and kinetics. One ancillary sample event for the activated sludge basin was conducted as part of an expanded methods analysis and included grab samples from Zones 1, 3, and 7, in addition to grab SE from the corresponding secondary clarifier.

At Facility 2, grab samples were collected weekly from the influent, CML effluent, and PML effluent for six consecutive weeks in the spring (March–April 2024) and six consecutive weeks in the summer (July–August 2024) (*N* = 12 total sample sets). The average daily air temperature ranges were 8°C–27°C for the spring sampling period and 27°C–40°C for the summer sampling period.

### 2.2 Sample Processing

Samples from Facility 1 were transported to the lab in coolers, inverted several times to mix, and immediately distributed into two 50‐mL conical tubes per sample. One conical tube was designated for bacteriophage plaque assays, and the second conical tube was designated for molecular analysis. As recommended by InnovaPrep (Drexel, MO), the molecular conical tube was supplemented with Tween‐80 (0.05% *v*/*v*) and agitated at 50 rpm for 10 min using an incubating orbital shaker to elute solids‐adsorbed viruses. After agitation, Facility 1 samples were centrifuged at ~3000 × *g* for 10 min (IEC Centra CL3R, Thermo Fisher Scientific, Waltham, MA). Approximately 50 mL of supernatant was then processed using the InnovaPrep Concentrating Pipette (CP) Select system with hollow fiber Concentrating Pipette Tips (CPTs; “Ultra” pore size; InnovaPrep). The concentrate was eluted with Tris‐based FluidPrep elution buffer (InnovaPrep), and the entire eluate was extracted into a final volume of 60 μL with a PureLink Viral RNA/DNA Mini Kit (Thermo Fisher Scientific). cDNA synthesis was then performed on 10 μL of extract using a Maxima First Strand cDNA Synthesis Kit (Thermo Fisher Scientific), resulting in a final cDNA volume of 60 μL. For the zone‐specific grab samples, the final cDNA was also diluted 10‐fold to reduce inhibition. Starting and processed volumes were recorded at each step for subsequent equivalent sample volume (ESV) calculations (described later).

For Facility 2, the molecular conical tube was centrifuged at ~3000 × *g* for 10 min, and the supernatant was processed using Amicon Ultra‐15 centrifugal filter units (MilliporeSigma, Burlington, MA) rather than the InnovaPrep CP Select system. This adjustment was necessary due to high residual turbidity in the Facility 2 supernatant, which clogged the InnovaPrep CPTs. Each Amicon centrifugal filter was used to process 30 mL of sample (one Amicon × two 15‐mL aliquots) down to ~500 μL of concentrate. Up to 350 μL of concentrate was extracted with a PureLink Viral RNA/DNA Mini Kit (Thermo Fisher Scientific), yielding a constant final elution volume of 60 μL. cDNA synthesis was then performed on 10 μL of extract using a Maxima First Strand cDNA Synthesis Kit (Thermo Fisher Scientific), resulting in a final cDNA volume of 60 μL.

To serve as a recovery proxy for the molecular analyses, 50 μL of attenuated bovine coronavirus (BCoV) stock solution (Zoetis Animal Health, Parsippany, NJ) was added to each 50‐mL sample from Facility 1. The BCoV stock solution was prepared by resuspending lyophilized BCoV with 1 mL of TE buffer (Sigma, St. Louis, MA), followed by a 1:50 dilution in TE. BCoV was not spiked into Facility 2 samples, so concentrations were not adjusted for recovery. In effect, similar recovery was assumed for all samples, which would result in the same calculated LRVs with or without recovery correction.

### 2.3 Virus Quantification

#### 2.3.1 Molecular: qPCR

For both DNA and RNA targets, cDNA extracts were analyzed in triplicate qPCR reactions using a CFX Opus 384 (Bio‐Rad, Hercules, CA), with 1 μL of template in a 10‐μL total reaction volume. The 1‐μL template volume resulted in a constant ESV of 0.14 mL (or 0.014 mL after 10‐fold dilution) for Facility 1 samples processed with the InnovaPrep CP Select system. For Facility 2, Amicon concentrate volumes varied (410 ± 409 μL), and only up to 350 μL was extracted, thereby resulting in variable ESVs (0.070 ± 0.023 mL).

Molecular targets included assays specific for enteric viruses (AdV 40/41, EnV, NoV GIA, and NoV GII), fecal indicator viruses (PMMoV, CGMMV, MS2 bacteriophage, and crAssphage), and BCoV as a surrogate for virus recovery. Primers, probes, and thermocycling details are provided in Table S1, and additional details on molecular methods are included in Text S1. Average gene copy (gc) counts across all replicates with successful amplification were converted to concentrations (in units of gc/L) using standard curves, sample‐specific ESVs, and sample‐specific BCoV recovery (Facility 1 only).

#### 2.3.2 Culture: Plaque Assays

Plaque assays were performed using the double agar layer method to quantify F‐specific and somatic coliphages using bacterial hosts *Escherichia coli* 15597 and *E. coli* 13706 (ATCC, Manassas, VA), respectively. Host cultures were grown in tryptic soy broth at 37°C with shaking at 150 rpm in an incubating orbital shaker until reaching log phase (OD_600_ ≈ 0.4–0.6). Samples were serially diluted in phosphate‐buffered saline (PBS) in 1‐mL microcentrifuge tubes to generate a range of concentrations appropriate for quantification. For each dilution, 100 μL of the prepared sample was mixed with 500 μL of the appropriate host culture in 5 mL of molten soft agar (0.7% tryptic soy agar [TSA] kept at 54°C). The mixture was poured onto TSA plates and incubated at 37°C for 16–24 h. Plaques were visually inspected and counted after incubation and then reported as plaque‐forming units per liter (PFU/L) after adjusting for sample volume and dilution factor, when applicable. All target dilutions were assayed in triplicate, and each set of experiments included positive and negative controls for the two bacterial hosts.

## 3 Results and Discussion

### 3.1 Facility 1: Conventional Activated Sludge System

#### 3.1.1 Composite Primary and Secondary Effluent

Based on water quality data provided by Facility 1 staff, the secondary biological treatment system achieved its primary goals in terms of BOD reduction (ΔBOD = 98 ± 0.9%), full nitrification (NH_3,SE_ = 0.07 ± 0.01 mg‐N/L), partial denitrification (ΔTN = 53 ± 3.0%), and phosphorus removal (ΔTP = 90 ± 1.7%) (Table 1). The MLSS concentration in the Facility 1 activated sludge system was 3154 ± 107 mg/L during the sampling period.

**TABLE 1:** General water quality data (mean ± 1 standard deviation) provided by facility staff for the Facility 1 primary effluent (PE) and secondary effluent (SE) (both composite) and the Facility 2 influent and effluent (both grab). For Facility 2, “effluent” reflects the regulatory compliance point (post‐chlorination), which is downstream of the partial mix lagoon (PML) effluent used for virus analysis.

Parameter	Units	Facility 1		Facility 2	
		PE	SE	Influent	Effluent
BOD	mg/L	184 ± 18	3.5 ± 1.5	218 ± 47	4.7 ± 3.5
TN	mg‐N/L	33 ± 2.2	16 ± 0.6	—	—
NH_3_	mg‐N/L	23 ± 2.3	0.07 ± 0.01	—	—
NO_2_ + NO_3_	mg‐N/L	2.1 ± 0.7	15 ± 0.9	—	—
TON^a^	mg‐N/L	8.1 ± 2.1	0.5 ± 0.8	—	—
TP	mg‐PO_4_/L	5.1 ± 0.6	0.5 ± 0.1	—	—
OPO_4_	mg‐PO_4_/L	2.3 ± 0.3	0.1 ± 0.0	—	—
TSS	mg/L	111 ± 24	11 ± 2.1	267 ± 45	20 ± 12

^a^ TON (calculated) = TN − NH_3_ − (NO_2_ + NO_3_).

Abbreviations: BOD = biochemical oxygen demand; OPO_4_ = orthophosphate; TN = total nitrogen; TON = total organic nitrogen; TP = total phosphorus; TSS = total suspended solids.

**FIGURE 1:**
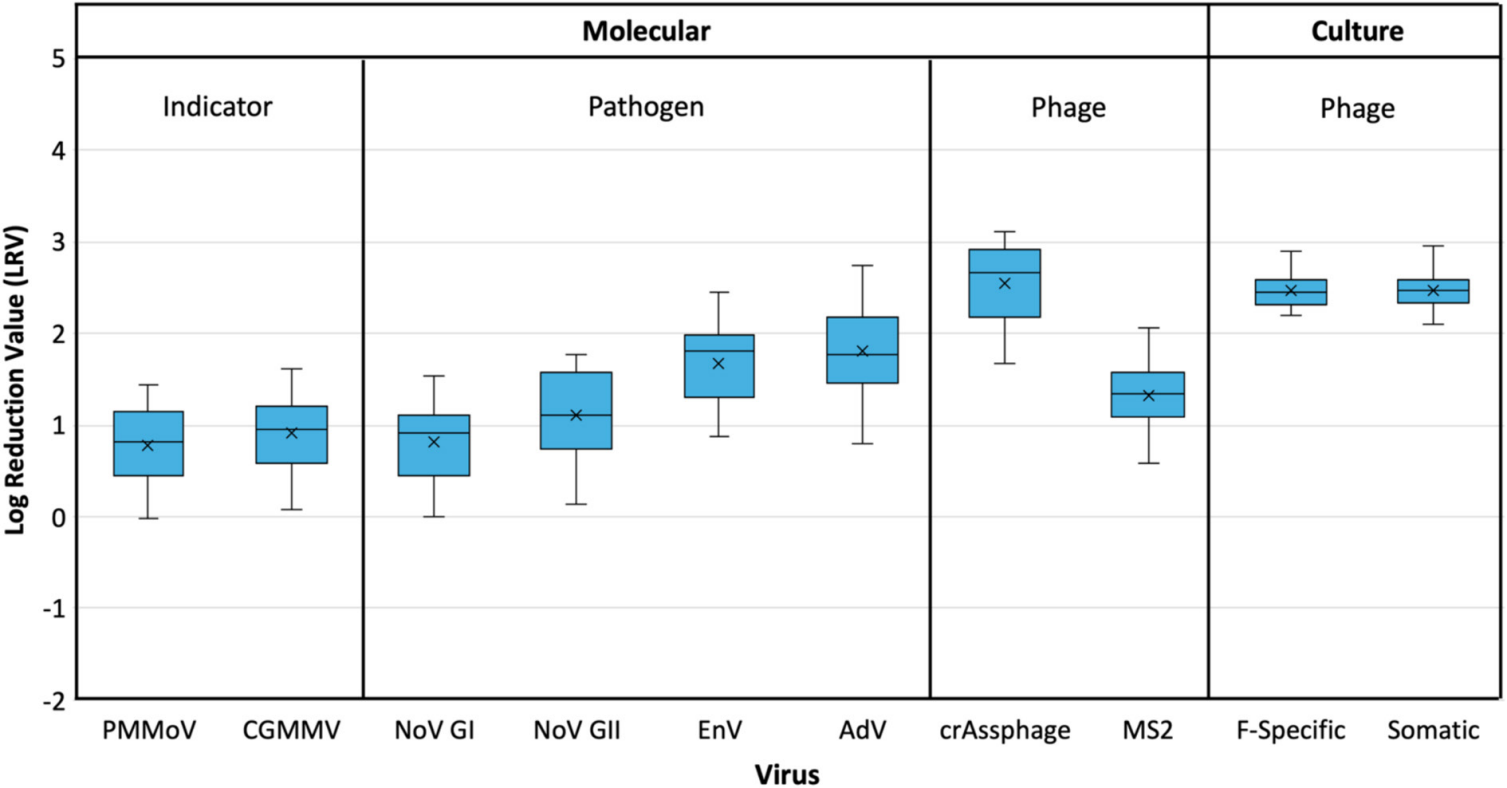
Box‐and‐whisker plots of virus log reduction values (LRVs) based on Facility 1 composite primary and secondary effluent samples. Each plot represents data from 32 to 36 paired composite samples (except for enterovirus with *N* = 23) and is presented in the Tukey style, with boxes indicating the 25th, 50th (median), and 75th percentiles; whiskers denoting ± 1.5 times the interquartile range (IQR); and the x symbols indicating means. The plots are separated based on virus type (indicator, pathogen, phage) and method of quantification (molecular, culture).

Across samples with successful qPCR‐based detection, BCoV recovery averaged 16 ± 17% and 24 ± 17% for primary (*n* = 34) and secondary effluent (*n* = 36), respectively; a summary of BCoV recovery is provided in Table S2. Recovery‐corrected molecular virus concentrations for the paired primary and secondary effluent samples are summarized in Table S3. These concentrations were used to generate virus‐specific LRVs for each sample pair, for which means are compared against corresponding literature values in Table 2 and distributions are illustrated as box‐and‐whisker plots in Figure 1. AdV and crAssphage exhibited the highest mean molecular LRVs, which is consistent with the bench‐scale SBR data in Wang et al. (2025) and within the range of molecular LRVs reported for AdV in the Hill et al. (2025) literature review. Based on ANOVA, the LRVs for NoV GI (*p* = 0.11) and GII (*p* = 0.72) were statistically similar across the three studies. Although the two fecal indicator plant viruses (PMMoV and CGMMV) yielded the lowest mean molecular LRVs in the current study and in the Wang et al. (2025) bench‐scale study, the full‐scale system performed better than the bench‐scale system for these viruses (*p* < 0.001).

**TABLE 2:** Comparison of mean (± 1 standard deviation) virus log reduction values (LRVs) across studies, in addition to published virus‐specific solids partitioning coefficients (*K*_d_).

Virus	Method	Current study (full‐scale)	Wang et al. (2025) (bench‐scale^a^)	Hill et al. (2025) (literature review)	Predicted (MLSS model^b^)	*K*_d_ (log_10_ mL/g) (Wang et al. 2025)
crAssphage	Molecular	2.6 ± 0.4	2.0 ± 0.9	—	1.7 ± 0.7	3.4 ± 0.8
Adenovirus	Molecular	1.8 ± 0.5	2.1 ± 0.8	2.3 ± 0.8^c^	2.0 ± 0.8	3.6 ± 1.0
Enterovirus	Molecular	1.7 ± 0.5	1.3 ± 0.4	2.2 ± 0.1^d^	1.4 ± 0.7	3.5 ± 0.8
MS2	Molecular	1.3 ± 0.4	0.8 ± 0.5	—	0.5 ± 0.4	2.5 ± 0.8
Norovirus GII	Molecular	1.1 ± 0.5	1.0 ± 0.4	1.1 ± 0.7	0.9 ± 0.5	3.0 ± 0.7
CGMMV	Molecular	0.9 ± 0.4	0.0 ± 0.6	—	0.3 ± 0.3	2.1 ± 0.8
Norovirus GI	Molecular	0.8 ± 0.4	1.1 ± 0.4	0.9 ± 0.6	0.9 ± 0.5	3.0 ± 0.7
PMMoV	Molecular	0.8 ± 0.4	0.4 ± 0.3	—	0.6 ± 0.4	2.6 ± 0.7
F‐specific coliphage^e^	Culture	2.5 ± 0.2	0.9 ± 0.8	1.8 ± 1.3	—	—
Somatic coliphage^e^	Culture	2.5 ± 0.2	1.5 ± 0.6	1.6 ± 0.5	—	—

^a^ Aggregated data for solids retention times (SRTs) of 7 and 20 days.

^b^ Predicted LRVs determined from 10,000 Monte Carlo simulations (see Section 3.1.1 for additional method details).

^c^ Combination of molecular (*N* = 45) and culture (*N* = 1) data.

^d^ Includes molecular data only (Hata et al. 2013).

^e^ F‐specific coliphage host = *E. coli* 15597 and somatic coliphage host = *E. coli* 13706.

To predict virus LRVs during secondary biological treatment, Wang et al. (2025) proposed an adsorption‐based physical removal framework incorporating virus‐specific solids partitioning coefficients (*K*_d_, mL/g) and MLSS concentrations (g/mL) (Equation 1). That study also demonstrated how the predictions could be improved by incorporating nucleic acid decay, although noting that nucleic acid decay might not be representative of virus inactivation.

(1) $$\mathrm{LRV}=\log_{10}\left(K_{d}\times \text{MLSS}+1\right)$$

Table 2 summarizes the predicted LRVs based on published *K*_d_ and decay parameters from Wang et al. (2025) coupled with the Facility 1 MLSS concentrations (3154 ± 107 mg/L). The predicted LRVs were calculated using a Monte Carlo approach with 10,000 simulations, assuming log_10_ normal distributions for virus‐specific *K*_d_ (Table 2) and median nucleic acid decay values (Wang et al. 2025). The prediction trends were consistent with the observed data, with higher LRVs for AdV, crAssphage, and EnV and lower LRVs for NoV, CGMMV, and PMMoV; MS2 is discussed in greater detail later. High LRVs were predicted and observed for viruses (e.g., AdV) that have been shown to exhibit extensive solids partitioning (i.e., high *K*_d_ value) and faster nucleic acid decay. Conversely, lower LRVs were predicted and observed for viruses (e.g., PMMoV) with a lower propensity for solids partitioning (i.e., low *K*_d_ value) and more stable nucleic acids. Finally, moderate LRVs were predicted and observed for viruses (e.g., NoV GII) with moderate solids partitioning and nucleic acid decay. Although consistent from a trend perspective, some viruses yielded higher observed LRVs in the current study than expected/predicted, namely CGMMV and crAssphage. In the case of CGMMV, its low predicted LRV was the result of minimal solids partitioning and decay observed in Wang et al. (2025). On the other hand, crAssphage exhibited high solids partitioning and decay in Wang et al. (2025), hence its high predicted LRV, but its observed LRV in the current study was even higher than expected. As described later, these unexpected results may be attributable to more extensive decay, at least for certain molecular targets, in this full‐scale system.

Despite consistency in trends and general agreement between observed and predicted values, virus LRVs in the current study and across the literature span large ranges, and the viruses relevant to public health (e.g., NoV, AdV, and EnV) often yield LRVs that dip below 1.0 at the lower percentiles. With respect to the fifth percentile LRVs that often drive regulatory determinations, AdV and EnV were ~1.0 based on molecular data in the current study, but NoV GI and GII were < 0.5, consistent with the literature (Hill et al. 2025). Moreover, Wang et al. (2025) reported particularly low LRVs for *culturable* AdV, which indicates that molecular data are not always a reliable surrogate for the culturable/infectious viruses that are more relevant to public health. In other words, molecular LRVs for some viruses may overestimate the level of public health protection achieved by secondary biological treatment.

This disconnect between molecular and culture LRVs may be due to high gene copy to infectious unit (GC:IU) ratios in influent wastewater—a scenario that has been demonstrated repeatedly for adenovirus (Crank et al. 2025; Pecson et al. 2021; Wang et al. 2025). This is the reason that nucleic acid decay had to be considered in the aforementioned MLSS model (Equation 1) when modeling molecular data. As an example, Figure S2 illustrates observed changes in GC:IU ratios from primary effluent to secondary effluent for AdV, EnV, and MS2. Figure S2 also shows that when GC:IU ratios remain relatively constant across secondary biological treatment, molecular and culture LRVs are more strongly correlated (Wang et al. 2025). Thus, particularly when nucleic acid decay (e.g., from nonculturable viruses) is more rapid than actual virus inactivation, efforts to eliminate this confounding signal in ‘upstream’ samples may be warranted. This was not explored in the current study, but future studies could evaluate whether enzyme‐mediated digestion or intercalating dyes could be used to improve correlations between molecular and culture LRVs. Also, it is important to emphasize that this nucleic acid decay is not necessarily representative of virus inactivation during secondary treatment. Incorporating nucleic acid decay improved model fit in Wang et al. (2025), but it should not be incorporated into a crediting framework unless it is explicitly linked to virus inactivation during secondary treatment.

In contrast with AdV, which appears to yield higher LRVs with molecular data (Wang et al. 2025), the molecular LRVs for MS2 in the current study were approximately half that of the culture LRVs for F‐specific coliphages (Figure 1 and Table 2). MS2 does not necessarily comprise all F‐specific coliphages, so this discrepancy could be related to non‐MS2 coliphages captured by *E. coli* 15597. The bench‐scale experiments in Wang et al. (2025) confirmed that different bacteriophages (T4, MS2, PR772, and phiX174) exhibit very different LRV profiles, including for direct comparisons of molecular and culture methods. Molecular assays targeting a broader spectrum of F‐specific coliphages (Wolf et al. 2008; Haramoto et al. 2009) may have exhibited greater consistency with the culture‐based data. Focusing on the culture data, the current study yielded surprisingly similar somatic and F‐specific coliphage LRVs (2.5 ± 0.2; Table 2). Importantly, differences in plaque morphology confirmed that the same host had not inadvertently been used for both assays, with larger plaques observed for *E. coli* 13706 and smaller plaques observed for *E. coli* 15597.

With respect to other full‐scale studies, Worley‐Morse et al. (2019) reported mean somatic and F‐specific coliphage LRVs ranging from ~1 to 2 and ~2 to 3, respectively, and Polanco et al. (2023) reported median somatic and F‐specific coliphage LRVs of ~2 and ~3, respectively. There is some consistency between these high LRVs for coliphages and those of EnV in the literature (Polanco et al. 2023; Wang et al. 2025), but they significantly overestimate LRVs for NoV (Wang et al. 2025). Thus, from a surrogate perspective, it is still unclear what role culturable coliphages might play in terms of crediting secondary biological treatment in potable reuse applications. Similar conclusions were reported by a meta‐analysis evaluating bacteriophages as operational monitoring tools (Amarasiri et al. 2017).

#### 3.1.2 Grab Samples From Activated Sludge Basin

**FIGURE 2:**
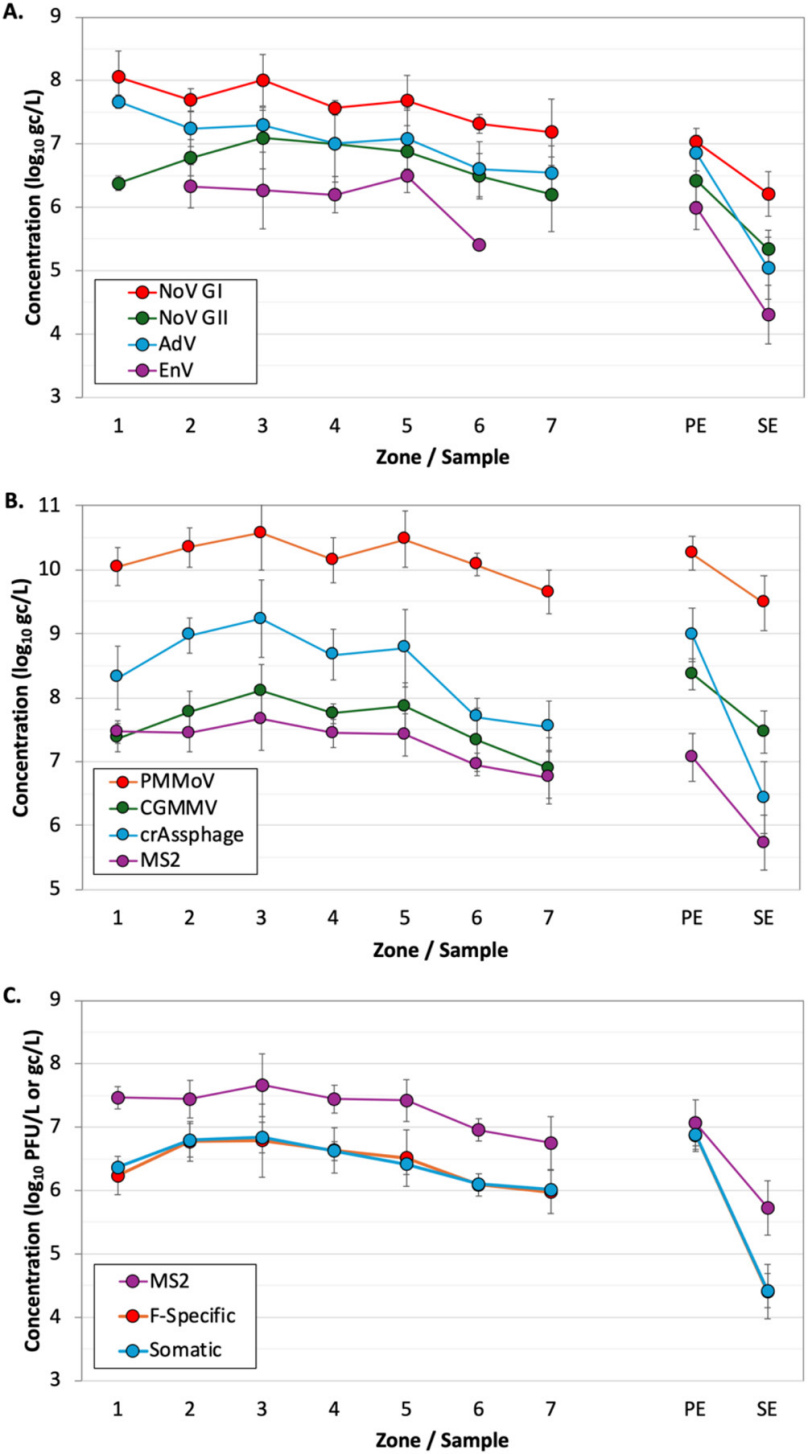
Concentrations of (A,B) pathogens, indicators, and phages quantified by qPCR (with recovery correction) and (C) phages quantified by culture methods, with molecular MS2 also shown for comparison. Data represent means (± 1 standard deviation) based on grab samples from the activated sludge basin (*N* = 1–3 per zone; see Figure S1 for zone schematic) or composite samples of the primary effluent (PE) or secondary effluent (SE) (*N* = 23–36 each). Molecular concentrations were determined in the sample supernatant after Tween‐80 addition and centrifugation, whereas culture concentrations were determined from direct plating (i.e., no solids removal).

To assess virus removal mechanisms through the activated sludge system of Facility 1, three sets of grab samples were collected from multiple zones within a single basin (illustrated in Figure S1). Concentrations for a majority of the viruses, including both molecular and culture data, often started lower in Zone 1 (anoxic) where RAS was introduced to the basin, and then concentrations increased in Zones 2–3 (anaerobic), presumably due to the introduction, mixing, and dispersion of primary effluent containing fresh viral loads (Figure 2). Once aeration commenced in Zone 4 and mixed liquor traveled through the remaining aerobic zones (5–7), virus concentrations generally decreased, albeit at a different rate for each virus. Virus concentrations were comparable between the grab mixed liquor from Zone 3, following the introduction of primary effluent, and the composite primary effluent from the earlier testing phase. The only two exceptions were NoV GI and AdV, which were significantly higher in the Zone 3 grab samples relative to the composite primary effluent (*p* = 0.05 for both).

The total viral load in each grab sample was determined to isolate the effect of decay/inactivation (relative to solids adsorption). For the molecular analyses, Tween‐80 was added to promote virus desorption from solids, followed by centrifugation for solids removal and nucleic acid extraction of the supernatant. However, culture samples were not treated with Tween‐80 and were instead plated directly (i.e., with solids included), after appropriate dilutions. To estimate how much viral decay occurred in the activated sludge basin (omitting physical removal), we compared total viral load in Zone 7 (basin outfall) versus Zone 3 (post‐introduction of primary effluent). This mass balance should theoretically reflect the overall level of decay that occurred in the activated sludge basin, which would then allow for a calculation of the first order decay rate constant. However, for the molecular targets, the measured viral load in Zone 7 was lower than expected. The ratio of the total viral load in Zone 7 versus Zone 3 ranged from 0.02 to 0.06 for crAssphage and CGMMV and up to 0.17 for AdV. While the AdV data are consistent, the crAssphage ratio is 10‐fold lower than what was observed in the corresponding bench‐scale dataset in Wang et al. (2025), which also reported no decay for CGMMV. That bench‐scale system was designed to mimic the full‐scale activated sludge system evaluated here. The mass balances on the other molecular targets were also similarly low in the current study.

To further investigate these low ratios, follow‐up samples were collected from multiple zones within the activated sludge basin and then analyzed with and without Tween‐80 addition for MS2, PMMoV, NoV GI, and NoV GII (Figure S3). Consistent with the original grab samples, concentrations increased from Zone 1 to Zone 3, decreased from Zone 3 to Zone 7, and then remained relatively constant from Zone 7 to the secondary effluent. Tween‐80 consistently increased virus concentrations except in the secondary effluent, presumably due to its lower solids content. However, the Zone 7 versus Zone 3 mass balances for the samples with Tween‐80 remained lower than previously reported. This suggests that Tween‐80 addition may not have adequately desorbed viruses (or nucleic acids) from the MLSS prior to centrifugation, and as aeration in the activated sludge basin promoted greater virus attachment to solids, they were subsequently removed to a greater extent during sample processing. Therefore, the grab samples and molecular analyses may not have provided an accurate total viral load for the corresponding mass balances, which could explain the discrepancies with Wang et al. (2025). The corresponding bench‐scale study employed a different nucleic acid extraction approach to differentiate solids and liquid partitioning, which may have led to a more accurate representation of total viral loads. For example, BCoV recovery was only 4.3 ± 3.1% for the activated sludge basin grab samples in the current study (Table S2) but was 63 ± 28% for comparable sample types in Wang et al. (2025). The higher BCoV recoveries in the earlier study (*p* < 0.001) suggest more reliable virus quantification. However, there was no direct measurement of BCoV adsorption and desorption from solids for the current study, which would be a more relevant measure to address this question.

In contrast with the molecular targets, culturable F‐specific and somatic coliphages were plated directly, without the confounding influence of Tween‐80 (Figure 2). While decay between Zone 3 and Zone 7 appeared to dominate the overall LRV for the molecular targets, decay (i.e., inactivation) explained only a portion of the overall LRV for the culturable coliphages. The observed coliphage LRV for Zone 7 relative to Zone 3 (inactivation without solids removal) was 0.7–0.8, but the earlier composite primary and secondary effluent samples (inactivation plus solids removal) indicated that the overall LRV for culturable coliphages was 2.5 ± 0.2 (Table 2). This suggests that physical removal may have been a more significant contributor to overall LRV, thereby justifying an expanded analysis.

**FIGURE 3:**
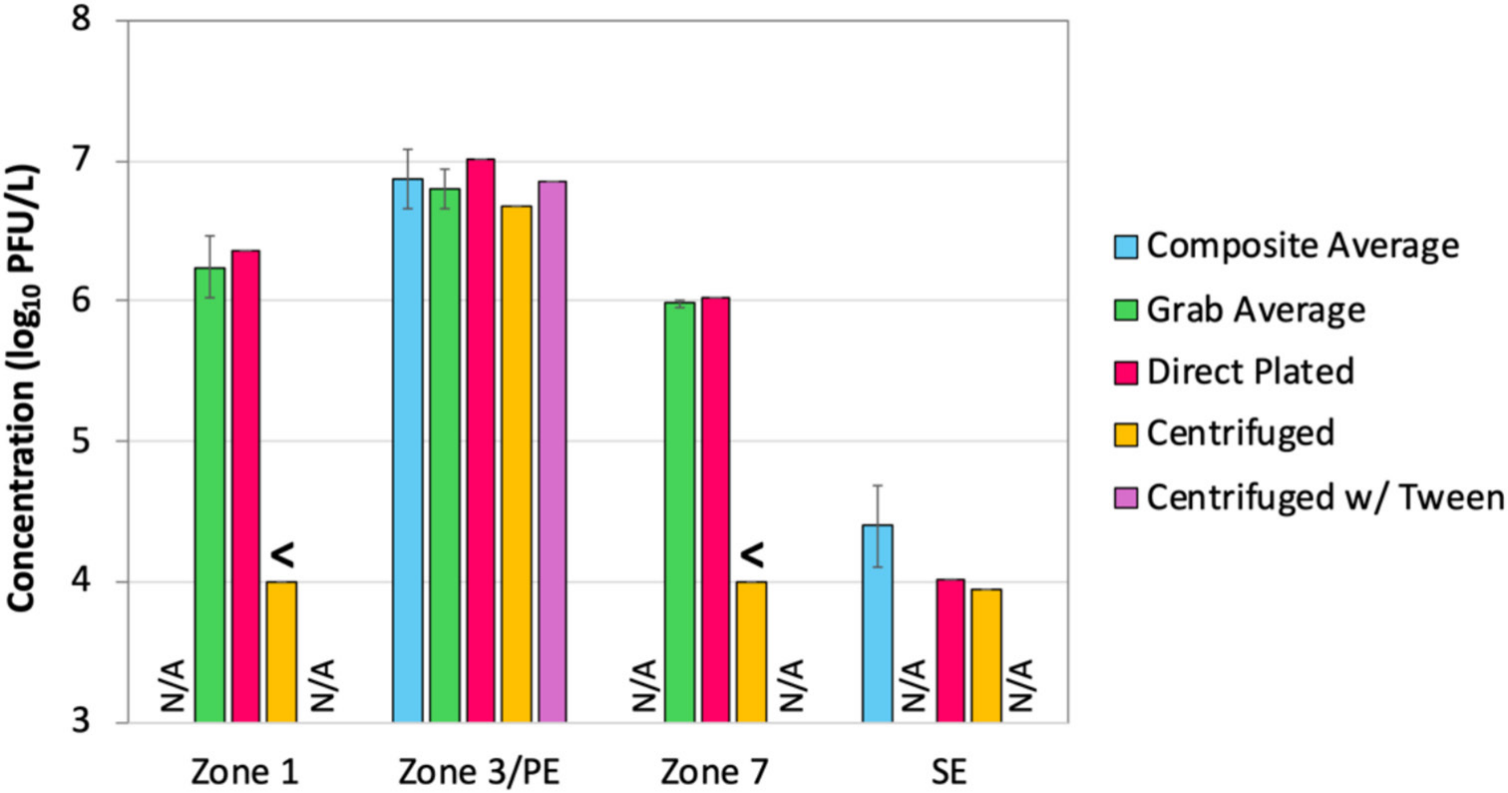
Culturable F‐specific coliphage concentrations for Facility 1. Data represent averages ± 1 standard deviation (when applicable). Concentrations are shown for the preceding 12‐week composite sample phase for primary effluent (PE) and secondary effluent (SE), the preceding 3‐week grab sample phase, and the follow‐up sampling of the activated sludge basin (direct plated, centrifuged, and centrifuged with Tween‐80). N/A = not applicable or not tested for that location; “<” indicates concentration was below the indicated limit of quantification.

As shown in Figure 3, the culture‐based data for F‐specific coliphages showed similar concentration trends as the molecular data, with a peak concentration in Zone 3 that presumably reflected the introduction of the primary effluent. Laboratory centrifugation of Zone 1 and Zone 7 samples resulted in dramatic drops in F‐specific coliphage concentration, suggesting significant adsorption of the F‐specific coliphages to the RAS in Zone 1 and the MLSS in Zone 7. In contrast, centrifugation of the Zone 3 sample resulted in a low LRV, suggesting that F‐specific coliphages introduced in the primary effluent had not yet adsorbed to the solids. Similarly, centrifugation also had a limited effect on the secondary effluent grab sample, presumably due to the more limited solids content rather than a lack of adsorption. Finally, the F‐specific coliphage concentration of the centrifuged Zone 7 sample was more consistent with the secondary effluent than the non‐centrifuged (i.e., directly plated) Zone 7 sample. For culturable coliphages, this suggests that simulated physical removal better reflected the secondary effluent than decay/inactivation alone.

### 3.2 Facility 2: Lagoon System

**FIGURE 4:**
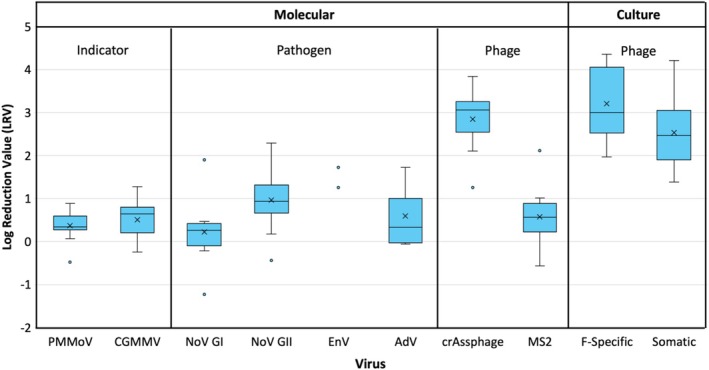
Box‐and‐whisker plots of virus log reduction values (LRVs) based on grab influent and grab partial mix lagoon (PML) effluent samples collected from Facility 2. Each plot represents data from 8 to 12 samples (except for enterovirus with *N* = 2 and adenovirus with *N* = 5) and is presented in the Tukey style, with boxes indicating the 25th, 50th (median), and 75th percentiles; whiskers denoting ± 1.5 times the interquartile range (IQR); circles representing outliers based on the IQR method; and the x symbols indicating means. The plots are separated based on virus type (indicator, pathogen, phage) and method of quantification (molecular, culture).

The Facility 2 samples were meant to provide a basis for comparison against the Facility 1 conventional activated sludge system, specifically to assess differences in removal mechanisms. Nonrecovery‐corrected virus concentrations for the grab influent, complete‐mix lagoon effluent, and partial‐mix lagoon effluent are summarized in Table S4. These concentrations were used to generate virus‐specific LRVs for each sample set, with the LRVs calculated based on the influent and PML effluent concentrations. The resulting LRV distributions are illustrated as box‐and‐whisker plots in Figure 4.

For most viruses, the molecular LRVs for the lagoon system exhibited similar trends but were slightly lower than those of the conventional activated sludge system at Facility 1. One notable difference was AdV, which yielded one of the higher average LRVs at Facility 1 (1.8 ± 0.5) but one of the lower average LRVs at Facility 2 (0.6 ± 0.8). As with Facility 1, the highest average LRVs were exhibited by molecular crAssphage (2.8 ± 0.6), F‐specific coliphages (3.2 ± 0.8), and somatic coliphages (2.5 ± 0.9). The somatic coliphage LRVs were similar between Facility 1 and Facility 2 (*p* = 0.78), albeit with greater variability in the lagoon system, but the F‐specific coliphage LRVs were higher on average for Facility 2 (*p* = 0.01). A review of wastewater pond systems reported a mean retention time of 14.5 days per log_10_ virus reduction (or base *e k* = 0.2 days^−1^) (Verbyla and Mihelcic 2015). The higher coliphage LRVs observed here, which equate to 1.8–3.9 days per log_10_ virus reduction (depending on phage and season), may be the result of the lagoon's surface aeration and/or relatively high temperatures, as described later.

It is important to note that because the Facility 2 molecular concentrations are not recovery corrected (i.e., no BCoV spike), relative differences in recovery between the influent and PML effluent could impact the calculated LRV. For example, if recovery was significantly lower for the Facility 2 influent, the actual influent concentrations would be higher than those described in Table S4, thereby augmenting the LRVs shown in Figure 4. That being said, recovery was similar for the primary and secondary effluent from Facility 1 (Table S2). Thus, even if the virus concentrations for the lagoon system are higher than reported, the corresponding LRVs might be unaffected. For Facility 2, this recovery uncertainty does not impact the culturable coliphage data because those samples were assayed directly.

**FIGURE 5:**
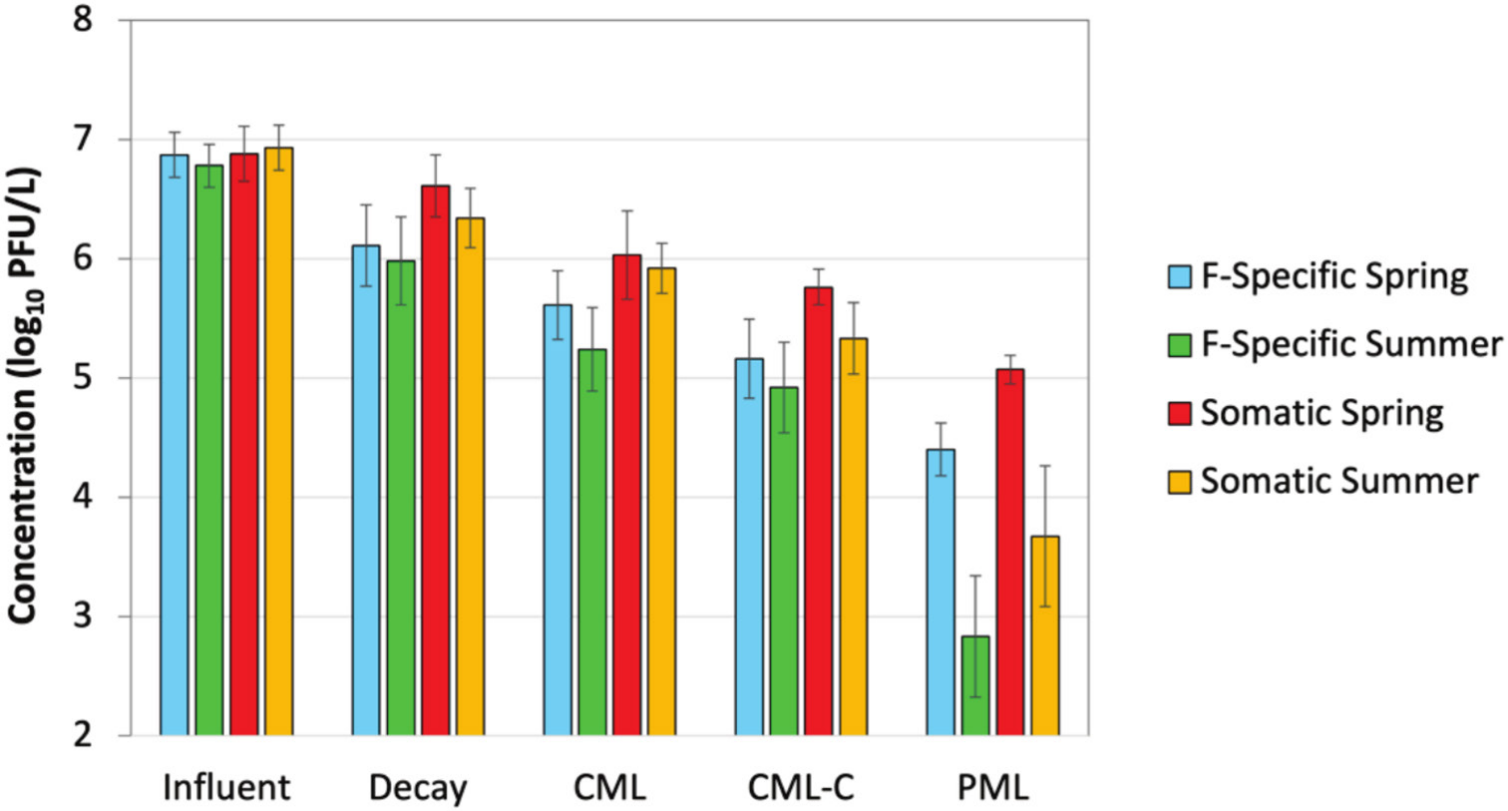
Culturable F‐specific and somatic coliphage concentrations for Facility 2. Data represent averages ± 1 standard deviation (when applicable). Concentrations (*N* = 6 per column) are divided by sampling location (influent, complete mix lagoon effluent [CML], and partial mix lagoon effluent [PML]), sample processing (direct plating vs. 7‐day influent decay or CML effluent plus centrifugation [CML‐C]), and season (spring vs. summer). For the decay analyses, influent samples were stored in the laboratory at room temperature for 7 days prior to plating.

Figure 5 illustrates changes in concentration of F‐specific and somatic coliphages as a function of season (spring vs. summer), sample location (influent vs. CML effluent vs. PML effluent), and sample processing (same‐day direct plating vs. 7‐day laboratory decay vs. centrifugation prior to plating to assess solids partitioning). For the influent, there was no difference in concentration for F‐specific versus somatic coliphages (*p* = 0.28) or when subdivided by season (*p* = 0.60). After holding the influent samples in the laboratory at room temperature for 7 days, specifically to mimic the overall HRT of the Facility 2 lagoon system, F‐specific coliphage concentrations decreased by ~0.8 log_10_ regardless of the season in which the samples were collected (base *e k* = 0.3 days^−1^), while somatic coliphage concentrations decreased by 0.3 log_10_ for the samples collected in spring and 0.6 log_10_ for the samples collected in summer (*k* = 0.1 and 0.2 days^−1^, respectively). These laboratory decay results are more similar to those reported in the review of lagoon systems (Verbyla and Mihelcic 2015), again pointing to surface aeration and/or temperature as key operational variables in the full‐scale system.

Depending on the season, the CML with an HRT of ~2 days achieved LRVs of 1.3–1.5 for F‐specific coliphages and 0.9–1.0 for somatic coliphages, with slightly higher LRVs associated with higher air temperatures (spring = 8°C–27°C vs. summer = 27°C–40°C). The seasonal LRV differences were not statistically significant, however (*p* = 0.07 and *p* = 0.15, respectively). With centrifugation, the LRVs for the CML effluent increased only 0.3–0.6 log_10_. In contrast, centrifugation of the Zone 1 and Zone 7 samples from Facility 1 resulted in > 2‐log_10_ reductions in F‐specific coliphage concentrations (shown previously in Figure 3). Given the smaller change in concentration for the CML effluent after centrifugation, it appears that decay/inactivation was more of a driving factor than solids partitioning in the lagoon system. Finally, the PML with an HRT of ~5 days yielded additional virus reduction, achieving overall LRVs (relative to the influent) of 2.5–4.0 and 1.8–3.3 for F‐specific and somatic coliphages, respectively. For both phage types, the higher summer temperatures increased the overall LRV by a statistically significant ~1.5 log_10_ (*p* < 0.001). Based on these results, longer HRTs coupled with reduced mixing/aeration in lagoon systems appear to shift the dominant mechanism for virus reduction from physical removal to decay/inactivation. In addition, the results indicate that decay/inactivation is significantly impacted by season, presumably driven by temperature differences.

### 3.3 Insights and Future Directions

Recent efforts to better understand virus removal during secondary biological wastewater treatment include compiling a robust dataset from the literature (Hill et al. 2025), conducting controlled bench‐scale experiments to eliminate confounding factors associated with large‐scale studies (Wang et al. 2025), and attempting to validate important findings through full‐scale monitoring (current study). One important outcome has been the identification of operational parameters that affect treatment performance, namely longer SRTs, higher MLSS concentrations, and mixing/agitation to promote viral attachment to solids for subsequent removal. However, this appears to be a more significant outcome for conventional activated sludge systems, as opposed to lagoon systems in which decay may dominate the overall LRV for culturable viruses. In reference to the virus LRVs reported by Polanco et al. (2023), Wang et al. (2025) noted that coagulant and/or polymer addition (or higher concentrations of these chemicals) may further enhance this physical removal mechanism.

One underexplored factor is the stochastic nature of virus interactions with non‐host microorganisms during secondary biological treatment. For example, human viruses such as NoV and rotavirus bind to histo‐blood group antigens (HBGAs) on intestinal epithelial cells (Nordgren et al. 2016). However, they also interact with HBGA‐like molecules found on bacterial surfaces, including those of *Enterobacter cloacae* , *E. coli* , and *Lactobacillus* spp. (Amarasiri et al. 2016; Amarasiri and Sano 2019; Miura et al. 2013). These interactions may facilitate virus attachment to biomass and flocs via microbial intermediaries, ultimately enhancing their removal through solids settling. Bench‐scale data on bacteriophage removal provide additional insight. Culturable somatic phages like T4 and phiX174, which interact with bacterial receptors that are consistently expressed, exhibit higher LRVs than F‐specific MS2 and N‐specific PR772 (Wang et al. 2025), which depend on conjugative pili that are conditionally expressed. Moreover, plant viruses such as PMMoV and CGMMV, which gain entry into their hosts through damaged cells rather than receptor‐mediated means, would likely have weak associations with microbial cells. Consistent with this hypothesis, plant viruses are consistently the least attenuated virus type in activated sludge systems (Papp et al. 2020; Wang et al. 2025). The innate predisposition for microbial surface binding by each virus type may be linked to their solids partitioning coefficients (i.e., *K*_d_), and these variable *K*_d_ values may be a function of microbial community structure or operational conditions (Roldan‐Hernandez et al. 2024). Future studies could seek to quantify virus‐biomass interactions using labeled viruses or targeted binding assays to investigate how microbial community composition influences virus LRVs. Incorporating these insights into predictive models may improve the accuracy of virus removal estimates and support the development of more robust crediting frameworks for potable reuse.

In addition to these physical removal considerations, the current study highlights that variable decay—or potentially methods that do not accurately characterize decay—may also be a significant factor leading to discrepancies between studies. Furthermore, differences between molecular (i.e., nucleic acid) decay and inactivation of culturable viruses call into question the value of molecular data as a means of crediting secondary biological treatment for virus removal. Molecular data has been useful in better understanding solids partitioning (Wang et al. 2025) but has also confounded estimates of decay/inactivation (current study). Future use of enzyme‐mediated digestion or intercalating dyes may help strengthen correlations between molecular and culture data, particularly for viruses with high GC:IU ratios (e.g., adenovirus).

For regulatory crediting frameworks, bacteriophages are often used or at least proposed as surrogates for enteric viruses, specifically to leverage their reduced laboratory safety concerns, lower costs, and quicker turnaround times. However, bacteriophage‐host interactions are more complex than traditional classifications suggest, complicating host selection and the suitability of bacteriophages as enteric virus surrogates. For example, *E. coli* 15597, a derivative of *E. coli* K‐12, is classified as an F‐specific bacteriophage host. However, *E. coli* K‐12 carries a transposon insertion disrupting the *wbb*L gene, which is responsible for forming sugar linkages in the oligosaccharide chains (O‐antigen) of lipopolysaccharide, making it susceptible to T‐even type bacteriophages. Additionally, both *E. coli* K‐12 and *E. coli* 15597 express the *ompC* gene, which encodes the OmpC porin protein recognized by T4 (a somatic phage). Cross‐reactivity of T4 with *E. coli* hosts 11303 (designated as T4‐specific), 15597 (designated as F‐specific), and 13706 (designated as somatic) is shown in Figure S4. Moreover, wild‐type phage populations can exhibit genetic variability that broadens their host range. For instance, mutants of phiX174 (e.g., JACS‐K and phiXtB) can infect *E. coli* K‐12 (Bone and Dowell 1973; Cox and Putonti 2010) and likely *E. coli* 15597, despite laboratory strains of phiX174 being unable to do so. These examples of bacteriophage cross‐reactivity could explain why measured bacteriophage concentrations and LRVs are sometimes similar when using *E. coli* hosts designated for F‐specific and somatic phages (Figure 1 and Figure 4). Because these culture‐based bacteriophage assays may then capture a broad mixture of bacteriophage types, they may or may not accurately represent enteric viruses, which are more relevant to public health. Thus, culture‐based bacteriophage data may provide value in elucidating treatment mechanisms, as demonstrated in the current study, but direct culture‐based measurement of enteric viruses, as demonstrated in Wang et al. (2025), may be more appropriate for regulatory decision‐making.

## 4 Conclusions

Facility‐specific operational conditions in conventional activated sludge systems, including SRT, MLSS concentration, coagulant/polymer use, and potentially even microbial community structure, play important roles contributing to the efficacy of secondary biological treatment for virus attenuation. For lagoon systems in which virus inactivation can be a significant mechanism, hydraulic retention times and temperature are also important factors. Predictive models provide value in estimating virus LRVs, particularly when trying to broadly differentiate between virus types. However, the main components of these predictive models, specifically solids partitioning coefficients (*K*_d_) and decay rate constants, exhibit large variability that still cannot be fully explained. This results in correspondingly large LRV ranges, in addition to low LRVs (i.e., < 0.5 log_10_) for some enteric viruses that are relevant to public health protection in potable reuse systems (e.g., norovirus). Thus, a broad regulatory crediting framework for conventional activated sludge systems in potable reuse applications does not appear to be justified at this time. Exceptions include site‐specific studies (Polanco et al. 2023) and membrane bioreactor (MBR) applications that leverage high MLSS concentrations and incredibly effective solids removal (Branch et al. 2023). If the factors that influence virus *K*_d_ and first‐order decay can be fully elucidated, there may be opportunities for broad LRV crediting of conventional activated sludge systems. Supplementing molecular methods with enzyme‐mediated digestion or intercalating dyes to eliminate the confounding effects of nucleic acids associated with damaged/inactivated virions may aid in this effort.

## Author Contributions

**Phillip Wang:** methodology, formal analysis, investigation, data curation, writing – original draft. **Tyler Hill:** methodology, investigation, data curation. **Christina Morrison:** investigation, writing – review and editing. **Katherine Crank:** investigation, data curation, writing – review and editing. **Jacimaria Batista:** supervision. **Daniel Gerrity:** conceptualization, funding acquisition, supervision, writing – review and editing.

## Conflicts of Interest

The authors declare no conflicts of interest.

## Supporting information


**Figure S1:** Schematics of the two full‐scale water resource recovery facilities (WRRFs), including descriptions of the sample collection sites. *Source:* Adapted from Google Maps.
**Table S1:** Primers, probes, and thermocycling conditions for the molecular methods.
**Table S2:** Summary of bovine coronavirus (BCoV) recovery for various sample types. Recovery was not evaluated for Facility 2.
**Table S3:** Summary of recovery‐corrected virus concentrations for Facility 1 primary effluent and secondary effluent (all composite samples), including mean (±1 standard deviation), median, and maximum concentrations. Reported means are based only on detected concentrations in N out of a total of 36 samples (i.e., data for censored/failed samples omitted).
**Figure S2:** (Left) Comparison of log10‐transformed gene copy to infectious unit (GC:IU) ratios for adenovirus, enterovirus, and MS2. (Right) Linear relationships between molecular and culture log reduction values (LRVs) for adenovirus, enterovirus, and MS2. Stronger linear relationships between molecular and culture LRVs are observed for viruses with more similar GC:IU ratios in primary effluent (PE) and secondary effluent (SE). *Source:* Adapted from Wang et al. (2025).
**Figure S3:** Effect of Tween‐80 addition on Facility 1 activated sludge samples. Grab samples were collected from zone 1 (anoxic; return activated sludge inlet), zone 3 (anaerobic; postintroduction of primary effluent), zone 7 (aerobic basin outfall), and the corresponding secondary effluent (SE) outfall. Samples were processed without (−) and with (+) Tween‐80 addition (plus centrifugation for solids separation) prior to molecular analysis. Columns indicate recoverycorrected molecular concentrations for (A) MS2, (B) PMMoV, (C) NoV GI, and (D) NoV GII.
**Table S4:** Summary of non‐recovery‐corrected virus concentrations [mean (±1 standard deviation), median, maximum] for Facility 2 grab samples of influent, complete mix lagoon (CML) effluent, and partial mix lagoon (PML) effluent. Reported means are based only on detected concentrations in N out of a total of 12 samples (i.e., censored data omitted).
**Figure S4:** Cross‐reactivity of bacteriophage T4 with diverse bacterial hosts, including *E. coli* 11303 (designated as T4‐specific), *E. coli* 15597 (designated as F‐specific), and *E. coli* 13706 (designated as somatic). *Source:* Adapted from Wang et al. (2025).

## Data Availability

Data will be made available by the corresponding author on request.

## References

[wer70180-bib-0001] Amarasiri, M. , S. Hashiba , T. Miura , et al. 2016. “Bacterial Histo‐Blood Group Antigens Contributing to Genotype‐Dependent Removal of Human Noroviruses with a Microfiltration Membrane.” Water Research 95: 383–391. 10.1016/J.WATRES.2016.04.018. 27095709

[wer70180-bib-0002] Amarasiri, M. , M. Kitajima , T. H. Nguyen , S. Okabe , and D. Sano . 2017. “Bacteriophage Removal Efficiency as a Validation and Operational Monitoring Tool for Virus Reduction in Wastewater Reclamation: Review.” Water Research 121: 258–269. 10.1016/j.watres.2017.05.035. 28551509

[wer70180-bib-0003] Amarasiri, M. , and D. Sano . 2019. “Specific Interactions Between Human Norovirus and Environmental Matrices: Effects on the Virus Ecology.” Viruses 11, no. 3: 224. 10.3390/v11030224. 30841581 PMC6466409

[wer70180-bib-0004] Amoueyan, E. , S. Ahmad , J. N. S. Eisenberg , and D. Gerrity . 2019. “Equivalency of Indirect and Direct Potable Reuse Paradigms Based on a Quantitative Microbial Risk Assessment Framework.” Microbial Risk Analysis 12: 60–75. 10.1016/j.mran.2019.06.003.

[wer70180-bib-0005] Bone, D. R. , and C. E. Dowell . 1973. “A Mutant of Bacteriophage φX174 Which Infects *E. coli* K12 Strains: I. Isolation and Partial Characterization of φXtB.” Virology 52, no. 2: 319–329. 10.1016/0042-6822(73)90326-7. 18620154

[wer70180-bib-0006] Branch, A. , A. Salveson , N. Fontaine , Z. Hirani , and S. Trussell . 2023. Evaluation of Tier 3 Validation Protocol for Membrane Bioreactors to Achieve Higher Pathogen Credit for Potable Reuse. Water Research Foundation.

[wer70180-bib-0007] Cox, J. , and C. Putonti . 2010. “Mechanisms Responsible for a ΦX174 Mutant's Ability to Infect *Escherichia coli* by Phosphorylation.” Journal of Virology 84, no. 9: 4860–4863. 10.1128/jvi.00047-10. 20147402 PMC2863744

[wer70180-bib-0008] Crank, K. , K. Papp , C. Barber , et al. 2025. “Pathogen and Indicator Trends in Southern Nevada Wastewater During and After the COVID‐19 Pandemic.” Environmental Science: Water Research & Technology 11, no. 2: 262–280. 10.1039/d4ew00620h.

[wer70180-bib-0009] EPA . 2012. 2012 Guidelines for Water Reuse. United States Environmental Protection Agency.

[wer70180-bib-0010] EPA . 2017. Potable Reuse Compendium. United States Environmental Protection Agency.

[wer70180-bib-0011] Gerrity, D. , K. Crank , E. Steinle‐Darling , and B. M. Pecson . 2023. “Establishing Pathogen Log Reduction Value Targets for Direct Potable Reuse in the United States.” AWWA Water Science 5, no. 5: e1353. 10.1002/aws2.1353.

[wer70180-bib-0012] Haramoto, E. , M. Kitajima , H. Katayama , M. Asami , M. Akiba , and S. Kunikane . 2009. “Application of Real‐Time PCR Assays to Genotyping of F‐Specific Phages in River Water and Sediments in Japan.” Water Research 43, no. 15: 3759–3764. 10.1016/j.watres.2009.05.043. 19555992

[wer70180-bib-0013] Hata, A. , M. Kitajima , and H. Katayama . 2013. “Occurrence and Reduction of Human Viruses, F‐Specific RNA Coliphage Genogroups and Microbial Indicators at a Full‐Scale Wastewater Treatment Plant in Japan.” Journal of Applied Microbiology 114, no. 2: 545–554. 10.1111/jam.12051. 23170920

[wer70180-bib-0014] Hill, T. , P. Wang , A. Olivieri , J. Batista , and D. Gerrity . 2025. “Assessing the Basis for Regulatory Crediting of Virus LRVs for Secondary Biological Wastewater Treatment: A Systematic Review.” Water Research 271: 122886. 10.1016/J.WATRES.2024.122886. 39647311

[wer70180-bib-0015] Kim, T. D. , N. Shiragami , and H. Unno . 1995. “Development of a Model Describing Virus Removal Process in an Activated Sludge Basin.” Journal of Chemical Engineering of Japan 28, no. 3: 257–262. 10.1252/jcej.28.257.

[wer70180-bib-0016] Miura, T. , D. Sano , A. Suenaga , et al. 2013. “Histo‐Blood Group Antigen‐Like Substances of Human Enteric Bacteria as Specific Adsorbents for Human Noroviruses.” Journal of Virology 87, no. 17: 9441–9451. 10.1128/jvi.01060-13. 23804639 PMC3754087

[wer70180-bib-0017] Nordgren, J. , S. Sharma , A. Kambhampati , B. Lopman , and L. Svensson . 2016. “Innate Resistance and Susceptibility to Norovirus Infection.” PLoS Pathogens 12, no. 4: e1005385. 10.1371/journal.ppat.1005385. 27115484 PMC4845991

[wer70180-bib-0018] Papp, K. , D. Moser , and D. Gerrity . 2020. “Viral Surrogates in Potable Reuse Applications: Evaluation of a Membrane Bioreactor and Full Advanced Treatment.” Journal of Environmental Engineering 146, no. 2: 04019103. 10.1061/(asce)ee.1943-7870.0001617.

[wer70180-bib-0019] Pecson, B. , E. Darby , G. Di Giovanni , et al. 2021. Pathogen Monitoring in Untreated Wastewater. Water Research Foundation.

[wer70180-bib-0020] Polanco, J. A. , J. Safarik , J. S. Dadakis , C. Johnson , and M. H. Plumlee . 2023. “Enteric Virus Removal by Municipal Wastewater Treatment to Achieve Requirements for Potable Reuse.” PLOS Water 2, no. 9: 1–27. 10.1371/journal.pwat.0000052.

[wer70180-bib-0021] Ray, H. , K. Papp , L. Green , B. S. Tseng , E. Dickenson , and D. Gerrity . 2024. “DNA Origami: Thinking ‘Outside the Fold’ for Direct Integrity Testing of Membranes for Virus Removal in Potable Reuse Applications.” Environmental Science: Water Research & Technology 10, no. 9: 2188–2200. 10.1039/d4ew00285g.

[wer70180-bib-0022] Roldan‐Hernandez, L. , C. Van Oost , and A. B. Boehm . 2024. “Solid‐Liquid Partitioning of Dengue, West Nile, Zika, Hepatitis A, Influenza A, and SARS‐CoV‐2 Viruses in Wastewater From Across the USA.” Environmental Science: Water Research & Technology 11: 88–99. 10.1039/d4ew00225c.

[wer70180-bib-0023] Verbyla, M. E. , and J. R. Mihelcic . 2015. “A Review of Virus Removal in Wastewater Treatment Pond Systems.” Water Research 71, no. 15: 107–124. 10.1016/j.watres.2014.12.031. 25613410

[wer70180-bib-0024] Walker, T. , N. Boyle , B. D. Stanford , C. Owen , and P. G. Biscardi . 2020. “Full‐Scale Evaluation of Critical Control Points and Monitors at a Reuse Facility.” AWWA Water Science 2, no. 5: e1195. 10.1002/aws2.1195.

[wer70180-bib-0025] Wang, P. , T. Hill , C. Morrison , et al. 2025. “The Case for Credit: Toward a Mechanistic Model of Solids Partitioning and Virus Removal for Secondary Biological Wastewater Treatment.” Water Research 283: 123857. 10.1016/J.WATRES.2025.123857. 40424927

[wer70180-bib-0026] Wen, Q. , C. Tutuka , A. Keegan , and B. Jin . 2009. “Fate of Pathogenic Microorganisms and Indicators in Secondary Activated Sludge Wastewater Treatment Plants.” Journal of Environmental Management 90, no. 3: 1442–1447. 10.1016/j.jenvman.2008.09.002. 18977580

[wer70180-bib-0027] Wolf, S. , J. Hewitt , M. Rivera‐Aban , and G. E. Greening . 2008. “Detection and Characterization of F+ RNA Bacteriophages in Water and Shellfish: Application of a Multiplex Real‐Time Reverse Transcription PCR.” Journal of Virological Methods 149, no. 1: 123–128. 10.1016/j.jviromet.2007.12.012. 18280588

[wer70180-bib-0028] Worley‐Morse, T. , M. Mann , W. Khunjar , L. Olabode , and R. Gonzalez . 2019. “Evaluating the Fate of Bacterial Indicators, Viral Indicators, and Viruses in Water Resource Recovery Facilities.” Water Environment Research 91, no. 9: 830–842. 10.1002/wer.1096. 30848516 PMC6849880

