# Supplementary material for: Virus Log Reduction Values and Dominant Mechanisms in Full‐Scale Secondary Biological Wastewater Treatment Systems
Source: Water Environ Res. 2025 Sep 18;97(9):e70180. doi: 10.1002/wer.70180 (PMC12446742; doi:10.1002/wer.70180)
Supplement: Supplementary file 1 — Figure S1: Schematics of the two full‐scale water resource recovery facilities (WRRFs), including descriptions of the sample collection sites. Source: Adapted from Google Maps. Table S1: Primers, probes, and thermocycling conditions for the molecular methods. Table S2: Summary of bovine coronavirus (BCoV) recovery for various sample types. Recovery was not evaluated for Facility 2. Table S3: Summary of recovery‐corrected virus concentrations for Facility 1 primary effluent and secondary effluent (all composite samples), including mean (±1 standard deviation), median, and maximum concentrations. Reported means are based only on detected concentrations in N out of a total of 36 samples (i.e., data for censored/failed samples omitted). Figure S2: (Left) Comparison of log10‐transformed gene copy to infectious unit (GC:IU) ratios for adenovirus, enterovirus, and MS2. (Right) Linear relationships between molecular and culture log reduction values (LRVs) for adenovirus, enterovirus, and MS2. Stronger linear relationships between molecular and culture LRVs are observed for viruses with more similar GC:IU ratios in primary effluent (PE) and secondary effluent (SE). Source: Adapted from Wang et al. (2025). Figure S3: Effect of Tween‐80 addition on Facility 1 activated sludge samples. Grab samples were collected from zone 1 (anoxic; return activated sludge inlet), zone 3 (anaerobic; postintroduction of primary effluent), zone 7 (aerobic basin outfall), and the corresponding secondary effluent (SE) outfall. Samples were processed without (−) and with (+) Tween‐80 addition (plus centrifugation for solids separation) prior to molecular analysis. Columns indicate recoverycorrected molecular concentrations for (A) MS2, (B) PMMoV, (C) NoV GI, and (D) NoV GII. Table S4: Summary of non‐recovery‐corrected virus concentrations [mean (±1 standard deviation), median, maximum] for Facility 2 grab samples of influent, complete mix lagoon (CML) effluent, and partial mix lagoon (PML) effluent [file WER-97-e70180-s001.pdf]

Supplementary Information

Virus Log Reduction Values and Dominant Mechanisms in Full-Scale Secondary Biological Wastewater Treatment Systems

Phillip Wang[a+], Tyler Hill[a,b,c+], Christina Morrison[a], Katherine Crank[a], Jacimaria Batista[b], Daniel Gerrity[a*]

[a]Southern Nevada Water Authority, P.O. Box 99954, Las Vegas, NV 89193

[b]University of Nevada Las Vegas, Department of Civil and Environmental Engineering and Construction, 4505 S. Maryland Parkway, Las Vegas, NV 89154

[c]Black & Veatch, 8965 S. Eastern Ave. #325, Las Vegas, NV 89123

*Corresponding Author. Email: daniel.gerrity@snwa.com

[+]These authors contributed equally to this article.

Table of Contents

List of Supplementary Tables

Table S1. Primers, probes, and thermocycling conditions for the molecular methods 4

Table S2. Summary of BCoV recovery for various sample types 6

Table S3. Summary of recovery-corrected virus concentrations for Facility 1.............................. 7

Table S4. Summary of non-recovery-corrected virus concentrations for Facility 2 10

List of Supplementary Figures

Figure S1. Schematics of the two full-scale water resource recovery facilities 3

Figure S2. Comparison of GC:IU and molecular vs. culture-based LRVs 8

Figure S3. Effect of Tween addition on Facility 1 activated sludge samples 9

Figure S4. Cross-reactivity of bacteriophage T4 with diverse bacterial hosts 11

List of Supplementary Texts

Text S1. Additional molecular methods 5

Text S2. References 12

Figure S1. Schematics of the two full-scale water resource recovery facilities (WRRFs), including descriptions of the sample collection sites. **Source:** Adapted from Google Maps.

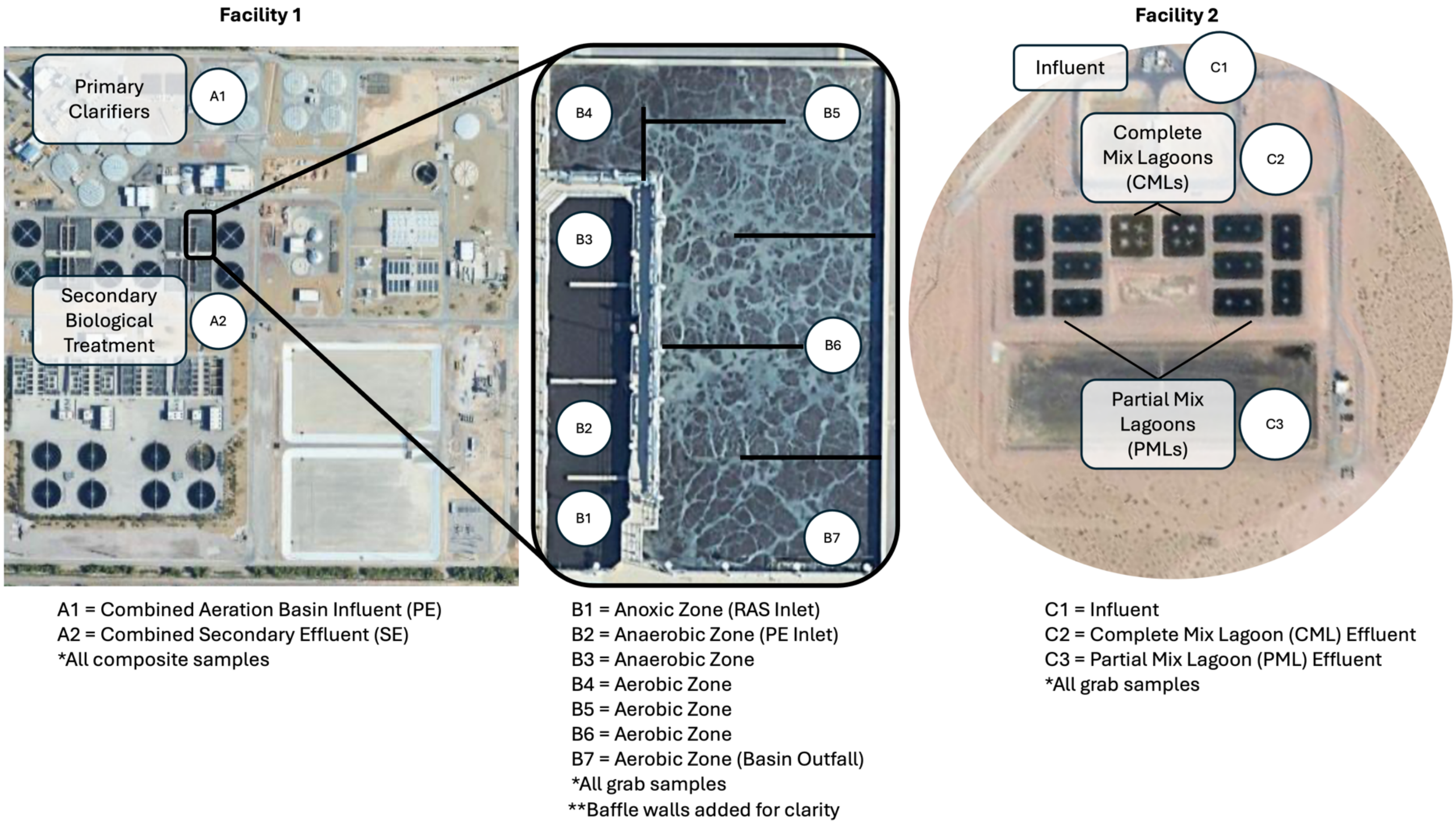

Table S1. Primers, probes, and thermocycling conditions for the molecular methods.

Target	Primer	Concentration (µM)	Cycling Condition	Reference
Adenovirus 40/41	F: AACTTTCTCTCTTAATAGACGCC	0.9	1 cycle: 2 min at 95°C,<br>40 cycles: 94°C for 30 sec and 57°C for 1 min	1,2
	R: AGGGGGCTAGAAAACAAAA	0.9		
	P: HEX-CTGACACGGGCACTCTTCGC-Zen/IBFQ	0.25		
Bovine Coronavirus	F: CTGGAAGTTGG TGGAGTT	0.9	1 cycle: 2 min at 95°C,<br>40 cycles: 94°C for 30 sec and 57°C for 1 min	3
	R: ATTATCGGCCTAACATACATC	0.9		
	P: HEX-CCTTCATATCTATACACATCAAGTTGTT-Zen/IBFQ	0.25		
crAssphage (CPQ_056)	F: CAGAAGTACAAACTCCTAAAAAACGTAGAG	0.9	1 cycle: 2 min at 95°C,<br>40 cycles: 94°C for 30 sec and 57°C for 1 min	4
	R: GATGACCAATAAACAAGCCATTAGC	0.9		
	P: FAM-AATAACGATTTACGTGATGTAAC-Zen/IBFQ	0.25		
Enterovirus	F: CCTCCGGCCCCTGAATG	0.9	1 cycle: 2 min at 95°C,<br>40 cycles: 94°C for 30 sec and 57°C for 1 min	2,5
	R: ACCGGATGGCCAATCCAA	0.9		
	P: FAM-CGGAACCGACTACTTTGGGTGTCCGT-Zen/IBFQ	0.25		
Norovirus GIA	F: CCATGTTCCGTTGGATGC	0.9	1 cycle: 2 min at 95°C,<br>40 cycles: 94°C for 30 sec and 57°C for 1 min	2,6,7
	R: TCCTTAGACGCCATCATCAT	0.9		
	P: Hex-AGATYGCGITCICCTGTCCA-Zen/IBFQ	0.25		
Norovirus GII	F: ATGTTCAGRTGGATGAGRTTCTCWGA	0.9	1 cycle: 2 min at 95°C,<br>40 cycles: 94°C for 30 sec and 57°C for 1 min	2,5
	R: TCGACGCCATCTTCATTCACA	0.9		
	P: FAM-AGCACGTGGGAGGGCGATCG-Zen/IBFQ	0.25		
PMMoV	F: GAGTGGTTTGACCTTAACGTTTGA	0.9	1 cycle: 2 min at 95°C,<br>40 cycles: 94°C for 30 sec and 57°C for 1 min	8,9
	R: TTGTCGGTTGCAATGCAAGT	0.9		
	P: FAM-GAGAGGCCT ACCGAAGCAAATGTCGC-Zen/IBFQ	0.25		
CGMMV	F:GCATAGTGCTTTCCCGTTCAC	0.9	1 cycle: 2 min at 95°C,<br>40 cycles: 94°C for 30 sec and 57°C for 1 min	10
	R:TGCAGAATTACTGCCCATAGAAAC	0.9		
	P: FAM-CGGTTTGCTCATTGGTTTGCGGA-Zen/IBFQ	0.25		
MS2	F:GTCCATACCTTAGATGCGTTAGC	0.9	1 cycle: 2 min at 95°C,<br>40 cycles: 94°C for 30 sec and 57°C for 1 min	11
	R:CCGTTAGCGAAGTTGCTTGG	0.9		
	P: FAM-CGACAATGGCGGAACTGGCGACGT-Zen/IBFQ	0.25		

Text S1. Supplemental molecular methods

Standard curves for qPCR were generated using known concentrations of serially diluted gBlock gene fragments from Integrated DNA Technologies (IDT, Newark, NJ). The concentration range for each standard curve was optimized to cover the expected range of sample concentrations. Standard curves were prepared in triplicate for each assay, with a minimum of five dilution points spanning five to six orders of magnitude. The efficiency of the qPCR assay was assessed by calculating the slope of the standard curve, with an efficiency range of 90-110% considered acceptable. A linear regression model was used to evaluate the relationship between the log of the dilution and the corresponding cycle of quantification (Cq). The coefficient of determination (R^2) for all standard curves was required to be ≥0.95 to ensure reliable quantification.

For quality control, no-template controls (NTCs) were included in each qPCR run to monitor potential contamination. To ensure reproducibility, standards and samples were run in triplicate for each assay. Inhibition testing was performed using nucleic acid extracts and their corresponding 10-fold dilution. Samples were considered free of inhibition if the diluted sample exhibited the expected change in Cq of 3.33±1, indicating no interference with the qPCR assay. This confirmed that the sample matrix did not affect amplification efficiency, thereby ensuring reliable and accurate quantification in subsequent analyses. Finally, as described in Borchardt et al. (2021), the Cq cut-off approach was used to establish a limit of quantification for this study (Cq = 38).

Table S2. Summary of bovine coronavirus (BCoV) recovery for various sample types. Recovery was not evaluated for Facility 2.

Facility	Sample Type	N	Mean	Min	Median	Max
1	Composite Primary	34	16 ± 17%	1.5%	10%	83%
1	Composite Secondary	36	24 ± 22%	1.8%	18%	106%
1	Grab Zone 1	2	1.5 ± 0.7%	1.0%	1.5%	1.9%
1	Grab Zone 2	3	2.6 ± 0.8%	2.0%	2.1%	3.5%
1	Grab Zone 3	3	3.9 ± 3.2%	0.7%	3.9%	7.0%
1	Grab Zone 4	3	3.3 ± 1.6%	1.6%	3.4%	4.9%
1	Grab Zone 5	2	5.2 ± 4.9%	1.7%	5.2%	8.7%
1	Grab Zone 6	2	6.7 ± 4.1%	3.8%	6.7%	9.6%
1	Grab Zone 7	2	8.6 ± 2.0%	7.2%	8.6%	10%

Table S3. Summary of **recovery-corrected** virus concentrations for Facility 1 primary effluent and secondary effluent (all composite samples), including mean (±1 standard deviation), median, and maximum concentrations. Reported means are based only on detected concentrations in N out of a total of 36 samples (i.e., data for censored/failed samples omitted).

Virus	Units	Primary Effluent				Secondary Effluent			
		N	Mean	Median	Maximum	N	Mean	Median	Maximum
PMMoV	$\log_{10}$ gc/L	35	10.3 ± 0.3	10.3	10.7	36	9.5 ± 0.4	9.4	10.2
CGMMV	$\log_{10}$ gc/L	35	8.4 ± 0.4	8.5	9.0	36	7.5 ± 0.6	7.4	8.3
Norovirus GI	$\log_{10}$ gc/L	34	7.0 ± 0.2	7.1	7.3	35	6.2 ± 0.4	6.2	6.8
Norovirus GII	$\log_{10}$ gc/L	34	6.4 ± 0.3	6.5	6.9	33	5.3 ± 0.3	5.3	6.1
Enterovirus	$\log_{10}$ gc/L	35	6.0 ± 0.4	6.0	7.2	23	4.3 ± 0.5	4.1	5.3
Adenovirus	$\log_{10}$ gc/L	35	6.9 ± 0.3	6.9	7.5	35	5.0 ± 0.5	5.0	6.1
crAssphage	$\log_{10}$ gc/L	35	9.0 ± 0.3	9.0	9.5	36	6.4 ± 0.3	6.4	7.1
MS2	$\log_{10}$ gc/L	35	7.1 ± 0.4	7.1	7.8	36	5.7 ± 0.4	5.8	6.5
F-specific[a]	$\log_{10}$ PFU/L	36	6.9 ± 0.2	6.9	7.4	36	4.4 ± 0.3	4.4	4.9
Somatic[b]	$\log_{10}$ PFU/L	36	6.9 ± 0.2	6.8	7.4	36	4.4 ± 0.3	4.5	4.9

[a]Coliphage host: *E. coli* 15597
[b]Coliphage host: *E. coli* 13706

Figure S2. (Left) Comparison of $\log_{10}$-transformed gene copy to infectious unit (GC:IU) ratios for adenovirus, enterovirus, and MS2. **(Right)** Linear relationships between molecular and culture log reduction values (LRVs) for adenovirus, enterovirus, and MS2. Stronger linear relationships between molecular and culture LRVs are observed for viruses with more similar GC:IU ratios in primary effluent (PE) and secondary effluent (SE). **Source:** Adapted from Wang et al. (2025).

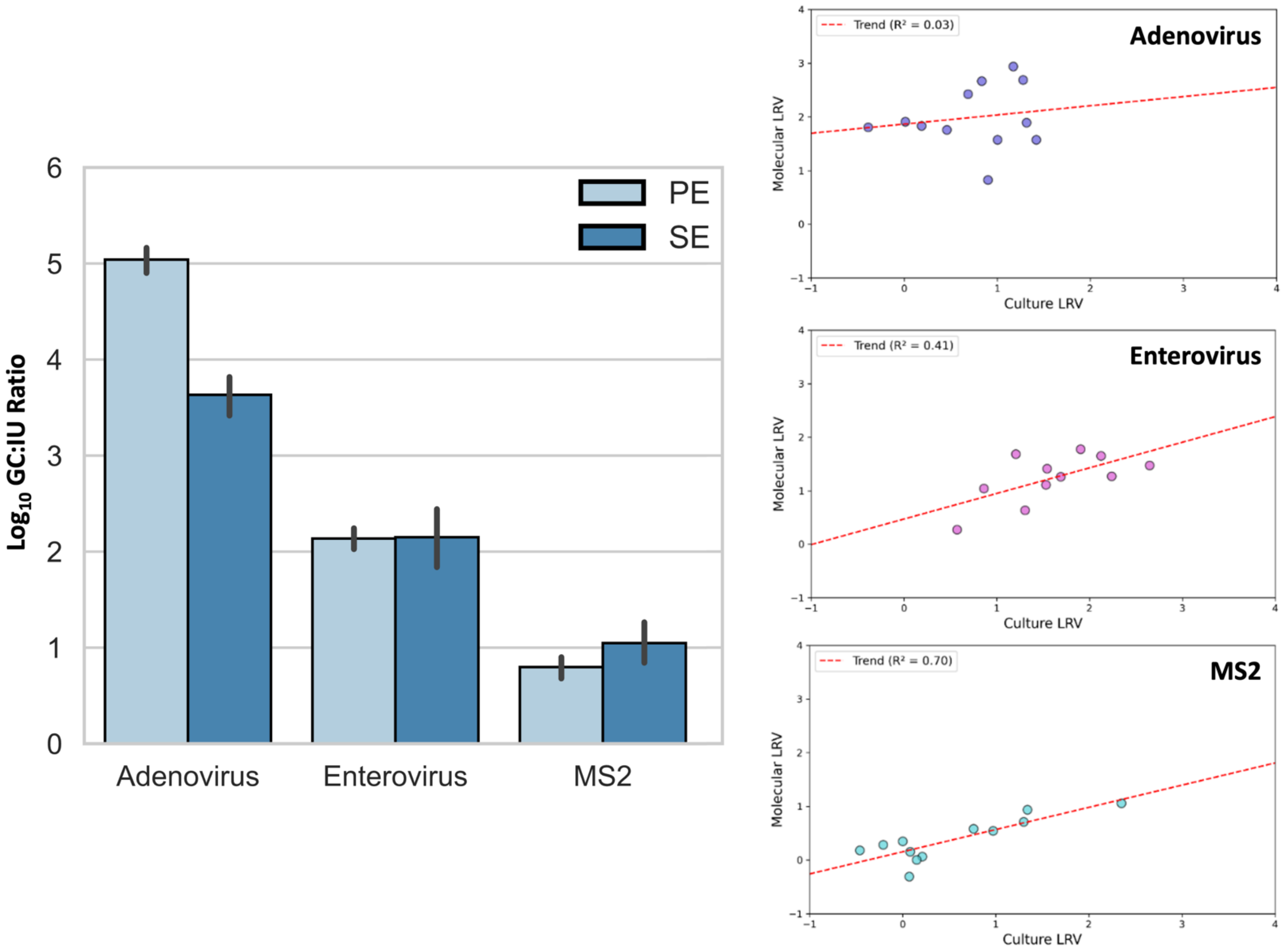

Figure S3. Effect of Tween-80 addition on Facility 1 activated sludge samples. Grab samples were collected from zone 1 (anoxic; return activated sludge inlet), zone 3 (anaerobic; post-introduction of primary effluent), zone 7 (aerobic basin outfall), and the corresponding secondary effluent (SE) outfall. Samples were processed without (-) and with (+) Tween-80 addition (plus centrifugation for solids separation) prior to molecular analysis. Columns indicate recovery-corrected molecular concentrations for **(A)** MS2, **(B)** PMMoV, **(C)** NoV GI, and **(D)** NoV GII.

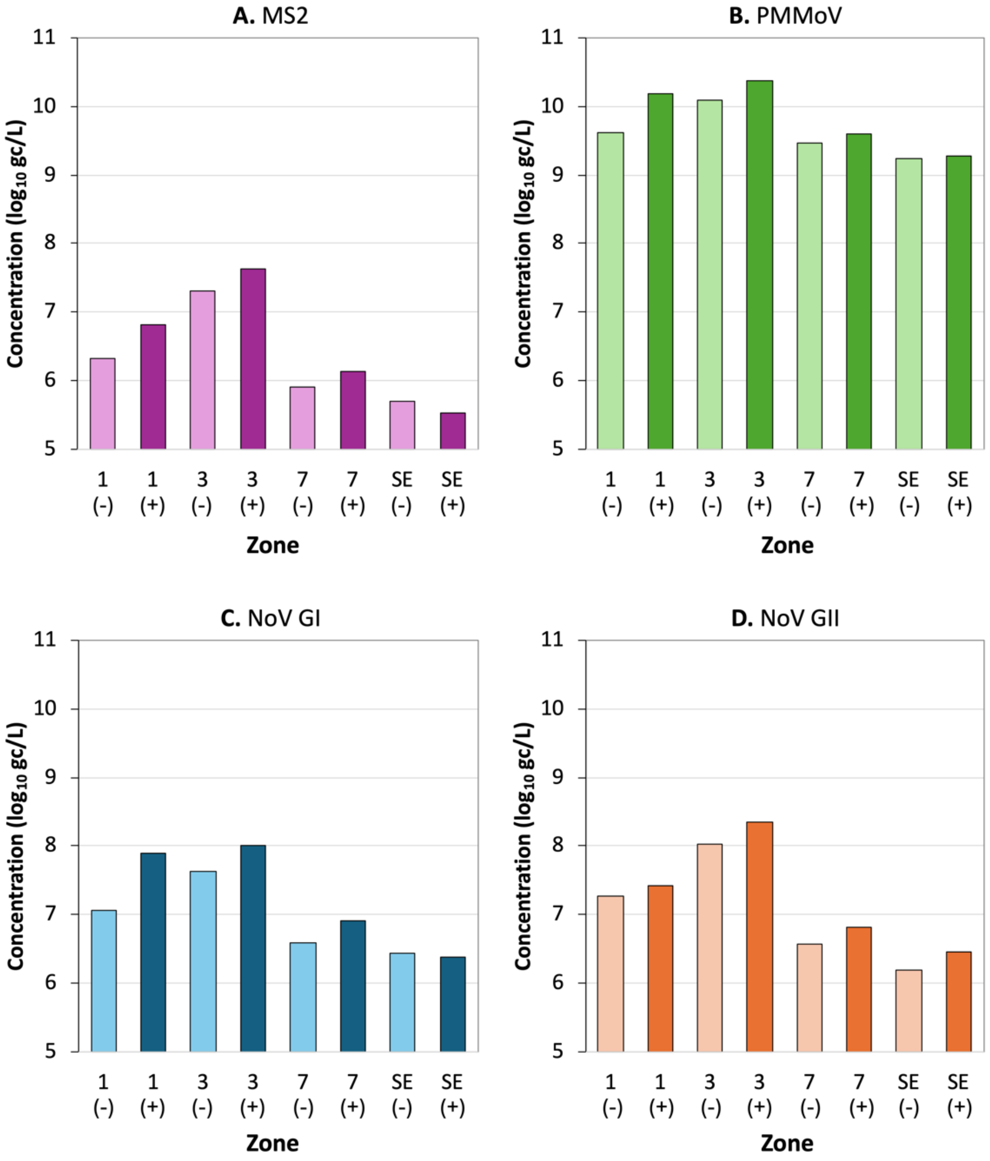

Table S4. Summary of **non-recovery-corrected** virus concentrations [mean (±1 standard deviation), median, maximum] for Facility 2 grab samples of influent, complete mix lagoon (CML) effluent, and partial mix lagoon (PML) effluent. Reported means are based only on detected concentrations in N out of a total of 12 samples (i.e., censored data omitted).

Virus	Units	Influent				CML Effluent				PML Effluent			
		N	Mean	Median	Max	N	Mean	Median	Max	N	Mean	Median	Max
PMMoV	log_{10} gc/L	12	8.9 ± 0.3	8.9	9.2	12	8.2 ± 0.8	8.5	9.3	12	8.5 ± 0.3	8.6	8.8
CGMMV	log_{10} gc/L	12	8.2 ± 0.6	8.3	9.4	12	7.4 ± 1.0	7.8	8.5	12	7.7 ± 0.4	7.8	8.2
Norovirus GI	log_{10} gc/L	9	6.1 ± 0.7	5.9	7.2	6	5.3 ± 0.5	5.3	5.9	8	5.9 ± 0.5	5.8	6.6
Norovirus GII	log_{10} gc/L	12	5.7 ± 0.4	5.6	6.2	9	4.9 ± 0.6	5.1	5.5	12	4.7 ± 0.5	4.7	5.5
Enterovirus	log_{10} gc/L	10	6.4 ± 0.8	6.2	7.6	3	4.7 ± 0.3	4.6	5.1	2	5.7 ± 0.2	5.7	5.8
Adenovirus	log_{10} gc/L	10	5.7 ± 0.6	5.4	7.0	6	4.6 ± 0.8	4.5	6.0	5	5.2 ± 0.6	4.9	6.1
crAssphage	log_{10} gc/L	12	9.0 ± 0.6	8.9	9.8	12	6.9 ± 1.0	6.9	8.6	12	6.2 ± 0.9	5.9	7.7
MS2	log_{10} gc/L	11	5.4 ± 0.8	5.7	6.5	12	5.0 ± 0.9	5.1	6.4	11	4.9 ± 0.9	4.9	6.3
F-specific[a]	log_{10} PFU/L	12	6.8 ± 0.2	6.9	7.1	12	5.4 ± 0.4	5.4	5.9	12	3.6 ± 0.9	3.9	4.6
Somatic[a]	log_{10} PFU/L	12	6.9 ± 0.2	6.9	7.2	12	6.0 ± 0.3	6.1	6.2	12	4.4 ± 0.8	4.6	5.3

[a]Coliphage host: *E. coli* 15597
[b]Coliphage host: *E. coli* 13706

Figure S4. Cross-reactivity of bacteriophage T4 with diverse bacterial hosts, including *E. coli* 11303 (designated as T4-specific), *E. coli* 15597 (designated as F-specific), and *E. coli* 13706 (designated as somatic). **Source:** Adapted from Wang et al. (2025).

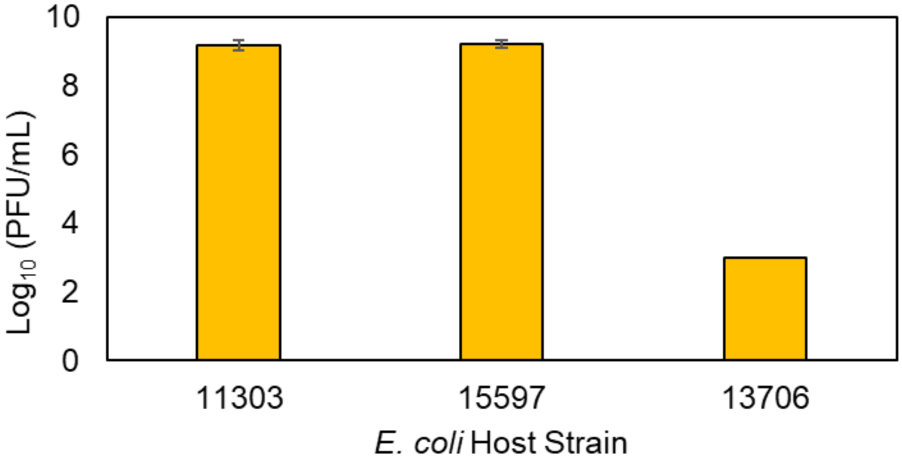

Text S2. References

1. Ko, G.; Jothikumar, N.; Hill, V. R.; Sobsey, M. D., 2005. Rapid detection of infectious adenoviruses by mRNA real-time RT-PCR. *Journal of Virological Methods*, *127*(2), 148-153.
2. Pecson, B. M.; Darby, E.; Danielson, R.; Dearborn, Y.; Di Giovanni, G.; Jakubowski, W.; Leddy, M.; Lukasik, G.; Mull, B.; Nelson, K. L, 2022. Distributions of waterborne pathogens in raw wastewater based on a 14-month, multi-site monitoring campaign. *Water Research*, *213*, 118170.
3. Decaro, N.; Elia, G.; Campolo, M.; Desario, C.; Mari, V.; Radogna, A.; Colaianni, M. L.; Cirone, F.; Tempesta, M.; Buonavoglia, C., 2008. Detection of bovine coronavirus using a TaqMan-based real-time RT-PCR assay. *Journal of Virological Methods*, *151*(2), 167-171.
4. Stachler, E.; Kelty, C.; Sivaganesan, M.; Li, X.; Bibby, K.; Shanks, O. C., 2017. Quantitative crAssphage PCR assays for human fecal pollution measurement. *Environmental Science & Technology*, *51*(16), 9146-9154.
5. EPA, 2012. Method 1615: Measurement of enterovirus and norovirus occurrence in water by culture and RT-qPCR. United States Environmental Protection Agency. 1-91.
6. Hill, V. R.; Mull, B.; Jothikumar, N.; Ferdinand, K.; Vinjé, J., 2010. Detection of GI and GII noroviruses in groundwater using ultrafiltration and TaqMan real-time RT-PCR. *Food and Environmental Virology*, *2*, 218-224.
7. Jothikumar, N.; Lowther, J. A.; Henshilwood, K.; Lees, D. N.; Hill, V. R.; Vinjé, J., 2005. Rapid and sensitive detection of noroviruses by using TaqMan-based one-step reverse transcription-PCR assays and application to naturally contaminated shellfish samples. *Applied and Environmental Microbiology*, *71*(4), 1870-1875.
8. Haramoto, E.; Kitajima, M.; Kishida, N.; Konno, Y.; Katayama, H.; Asami, M.; Akiba, M., 2013. Occurrence of pepper mild mottle virus in drinking water sources in Japan. *Applied and Environmental Microbiology*, *79*(23), 7413-7418.

9. Wang, P.; Hill, T.; Morrison, C.; Black, A.; Crank, K.; Mull, B.; Batista, J.; Gerrity, D., 2025. The case for credit: Toward a mechanistic model of solids partitioning and virus removal for secondary biological wastewater treatment. *Water Research*, in press.
10. Sivaganesan, M.; Haugland, R. A.; Chern, E. C.; Shanks, O. C., 2010. Improved strategies and optimization of calibration models for real-time PCR absolute quantification. *Water Research*, *44*(16), 4726-4735.
11. Turgeon, N.; Toulouse, M. J.; Martel, B.; Moineau, S.; Duchaine, C., 2014. Comparison of five bacteriophages as models for viral aerosol studies. *Applied and Environmental Microbiology*, *80*(14), 4242-4250.
12. Borchardt, M. A.; Boehm, A. B.; Salit, M.; Spencer, S. K.; Wigginton, K. R.; Noble, R. T., 2021. The environmental microbiology minimum information (EMMI) guidelines: qPCR and dPCR quality and reporting for environmental microbiology. *Environmental Science & Technology*, *55*(15), 10210-10223.
